# How important are working relationships for stroke self-management? A qualitative study with healthcare professionals in community stroke teams

**DOI:** 10.1177/02692155251358457

**Published:** 2025-07-21

**Authors:** Lauren Lucas, Sarah Peters, Sarah Cotterill, Audrey Bowen

**Affiliations:** 1 Manchester Centre for Health Psychology, School of Health Sciences, University of Manchester, Manchester, UK; 2 Northern Care Alliance NHS Trust, Greater Manchester, UK; 3 Geoffrey Jefferson Brain Research Centre & The Manchester Academic Health Science Centre, Manchester, UK; 4 Centre for Biostatistics, School of Health Sciences, 5292 University of Manchester, Manchester, UK

**Keywords:** Community rehabilitation, stroke, self-management, therapeutic alliance, qualitative study

## Abstract

**Objective:**

To understand healthcare professionals’ experiences of developing therapeutic alliances (working relationships) with stroke survivors, and their views on how alliance relates to self-management in community settings.

**Design:**

Qualitative study.

**Setting:**

Community.

**Participants:**

Healthcare professionals recruited purposively from four National Health Service community stroke teams in England.

**Main measures:**

Semi-structured, one-to-one qualitative interviews, transcribed verbatim and analysed using Braun and Clarke's reflexive thematic analysis.

**Results:**

Nineteen clinicians (six physiotherapists, four occupational therapists, two speech and language therapists, two nurses, one psychologist and four people in assistant/trainee roles) were included in the study. Three main themes were developed from the data. (1) *The team can’t come forever*: alliances were shaped by the time-limited nature of community rehabilitation and relied on trust, buy-in, and clearly defined roles and expectations. (2) *Therapeutic alliances help and hinder*: whilst alliances supported motivation and engagement, complicated power dynamics sometimes undermined self-management. (3) *Confusion about what self-management is*: participants often equated self-management with self-directed rehabilitation and described a lack of clarity, confidence and training in supporting emotional and long-term adjustment needs. Strong alliances were viewed as essential for self-management, but formal support strategies were rarely used.

**Conclusions:**

Community-based healthcare professionals consider therapeutic alliance to be the foundation for stroke self-management in the community. However, a limited understanding of self-management among clinicians, combined with unbalanced power dynamics, may restrict patient autonomy. Relationship-based training (e.g. Bridges) and the development of self-management champion roles within organisations may enhance clinicians’ confidence and consistency in delivering self-management support in the community.

## Introduction

Self-management is a key component of stroke rehabilitation, building confidence to manage stroke's life-long consequences.^1^ It relates to ‘*an individual's ability, in conjunction with family, community, & the appropriate healthcare professionals to manage symptoms, treatment, physical, psychosocial, cultural, & spiritual consequences & inherent lifestyle challenges required for living with a chronic disease’.*^2^^(p1145)^ Supported self-management includes behaviour change approaches, differing from self-directed rehabilitation which is the practice of therapy tasks away from the clinical setting.^1^ However, professionals’ varying interpretation of self-management support leads to inconsistent implementation in practice.^3^

Self-management is underpinned by several tasks and skills, one of which is the formation of a patient-provider partnership.^4,5^ This partnership, known as the therapeutic alliance, or working relationship, is comprised of the client/clinician bond, and agreement around the tasks/goals of therapy.^6^ It has long been viewed a catalyst for treatment success across many health populations.^7–9^

In stroke, alliance is an active component of the rehabilitation process and central for patient engagement and motivation.^10,11^ However, alliance research is in its infancy, mostly focused on single professions and inpatient settings.^10,11^ We know little about alliances in community settings or their influence on self-management. Poor alliance experiences can lead to disengagement from current and future episodes of care.^12^ In community mental health settings, the duration of the alliance positively^13,14^ or negatively^15^ affects self-management ability. The UK national service model for integrated community stroke services offers a ‘*seamless continuity of therapy’* for as long as patients are willing and able to participate, with options to self-refer.^16^ Given the nuances of therapeutic alliances in stroke,^17–20^ it is important to understand how alliances are developed with self-management and the current service delivery model in mind. Therefore, this research aims to understand healthcare professionals’ experiences of developing therapeutic alliances and their views on how alliance relates to self-management in community stroke rehabilitation.

## Methods

The study was approved by The North of Scotland (2) Research Ethics Committee (REC ref: 22/NS/0119). A phenomenological methodology, underpinned by critical realism, was adopted to understand the experiences of professionals developing therapeutic alliances with stroke survivors and how this relates to self-management.

The study was conducted in four National Health Service (NHS) Community Stroke Teams in England. Purposive sampling selected participants who met the following inclusion criteria and were judged to have sufficient knowledge and experience relevant to the research. (1) Registered or unregistered healthcare professionals working in a multidisciplinary Community Stroke Team; (2) at least 1-month clinical experience. The researcher (LL) liaised with a lead contact from each participating team, and then joined online team meetings to explain the research. An invitation flyer was distributed, and expressions of interest received by email and text. Eligible participants were provided with an information sheet and interviews were arranged. Participants provided written or verbal consent prior to being interviewed.

Semi-structured interviews were chosen because they offer flexibility.^21^ An interview schedule ensured data collected were grounded in answering the research questions, whilst also allowing participants to explore and address topics they considered important.^22^ This schedule was developed collaboratively by the authors and the project Lived Experience Group and was informed by existing research on therapeutic alliance in stroke^18,19^ and self-efficacy theory.^23^ A mock interview conducted prior to recruitment determined the clarity of questions and an approximate interview length. Interview options were flexible including online (Zoom/MS Teams), in-person, or on the telephone and were conducted during the participants’ working hours. In-person and telephone interviews were audio recorded and conducted in a private room, away from clinical areas to maintain confidentiality. Online interviews were video-recorded, un-disturbed and conducted alone, in a quiet room. Video-recorded interviews were transcribed by the first author and audio recorded interviews were sent to a university approved transcription service. Transcripts were not returned to participants to reduce time demand on NHS staff and to avoid the loss of valuable material if participants amended their original statements.^24^

Interviews were conducted by the first author (LL), an experienced physiotherapist in community stroke rehabilitation with a master's degree, who identifies as a woman. A comprehensive reflexive diary was maintained during the interview process documenting interviewing style, challenges and key topics and ideas to explore in subsequent interviews. Meetings with the research team, with backgrounds in neuropsychological rehabilitation and stroke (AB), healthcare communication and health psychology (SP), and health behaviour change and public health (SC), provided opportunity to discuss interviewing style, the layers of being an insider researcher^25^ and the impact of positionality on data interpretation.

Data were analysed using Braun and Clarke's six-stage reflexive thematic analysis,^26^ which began following the first participant interview. After 11 interviews recruitment was paused to conduct an in-depth analysis, adopting an experiential orientation to data interpretation, justified by the phenomenological nature of the research.^27^ Data were coded using NVivo 12 software by the first author (LL). Inductive, line by line coding of the 11 transcripts ensured respondent/data-based meanings were emphasised and generated initial semantic and latent codes, some of which were explored in the subsequent eight interviews.^27^ Some items of information were double coded according the semantic meaning communicated by the participant and the latent meaning interpreted by the researcher; however, no priority was given to either coding type.^27^ A selection of transcripts was discussed with the co-authors (AB, SP, SC) supporting the analysis process, sense checking both the semantic and latent meaning of the data and refining the interview topic guide. As the first round of open coding highlighted patterns in the codes and data, an interpretation of the aggregated meaning and meaningfulness across the dataset had already begun.^27^ Whilst open coding continued on the remaining eight transcripts, the analysis was influenced by the researcher having a deep understanding of the dataset and the existing narratives that were meaningful and relevant to the research question.^27^ An iterative process of generating and reviewing themes and sub-themes reviewed relationships between the data items and codes that informed them, and assessed how successfully they interpret the data in relation to the research question.^27^ Qualitative data were reported in line with the consolidated criteria for reporting qualitative research (COREQ) checklist.^28^

A group of seven public contributors formed a Lived Experience Group and were involved throughout the research pipeline. Their involvement helped develop study documentation and the interview schedule to support an ethics application. Following data analysis, the group provided feedback on the themes and sub-themes and helped to name them, ensuring the name aligned with the narrative of the data presented by the researcher (LL). Involving the group in this task provided opportunity to sense check the meaning of the data against the research questions, and generated recommendations for clinicians from people with lived experience of stroke, rehabilitation and self-management. These recommendations have helped to shape the clinical messages detailed in this paper.

## Results

**Figure 1.**
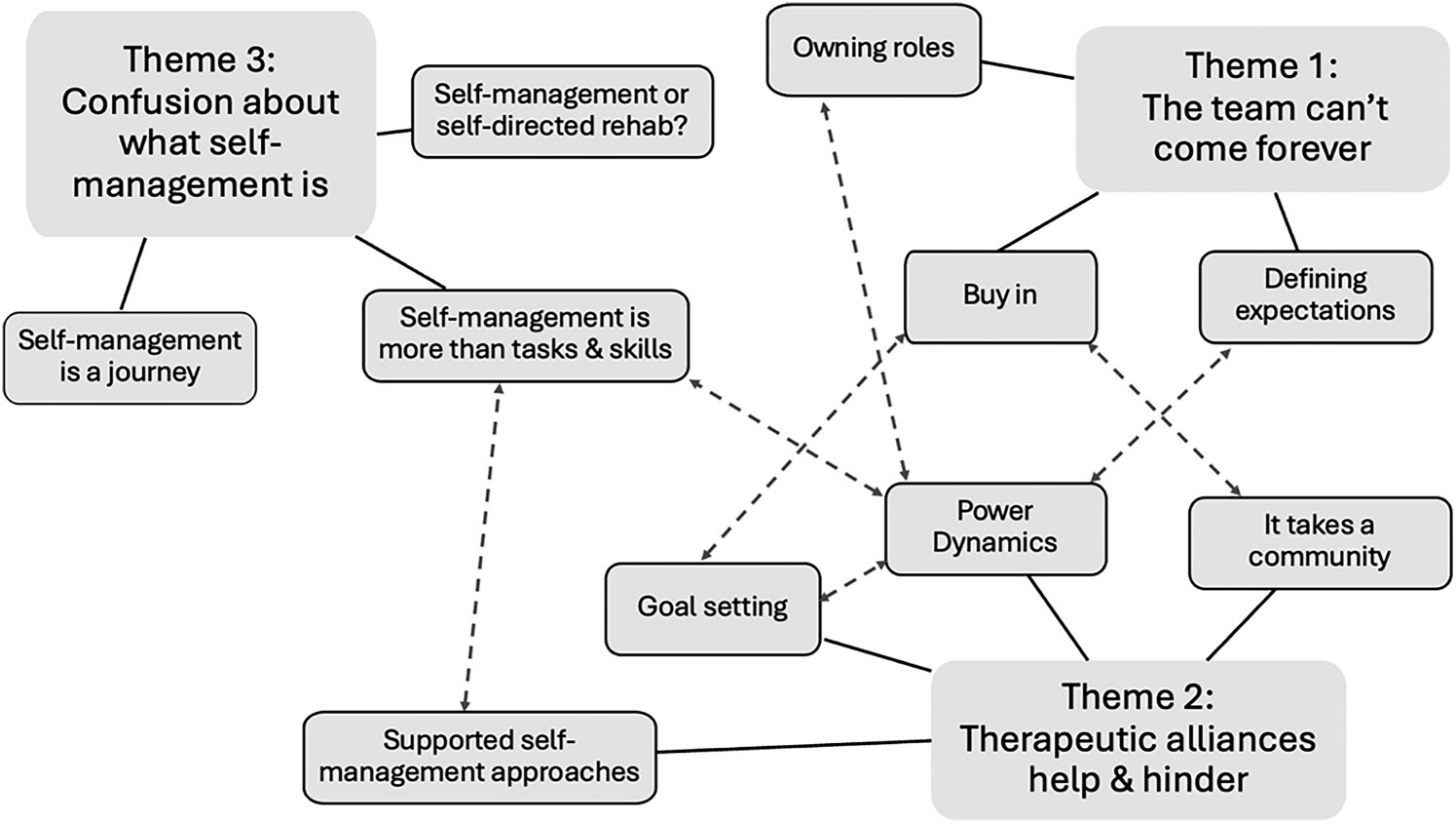
Thematic map detailing three themes and 10 sub-themes. A solid line represents how the sub-themes link to themes; a dashed line represents a relationship between sub-themes.

Nineteen healthcare professionals from four Community Stroke Teams were interviewed (Table 1). Interviews were conducted in-person (n = 9), online (n = 9), and on the telephone (n = 1), lasting between 30 and 90 min (mean = 60). Eight participants reported having completed self-management training, including self-directed reading, in-service training, local and national quality improvement initiatives, and as part of a university degree. The analysis led to three themes illustrated in Figure 1: (1) The team can’t come forever, (2) Therapeutic alliances help and hinder and (3) Confusion about what self-management is.

**Table 1.** Description of the sample and of the interviews.

Frequency (n = 19)		N (%)
Gender identity	Woman	14 (74)
	Man	5 (26)
Ethnicity	White British	18 (95)
	Mixed white and Asian	1 (5)
Age (years)	20–29	3 (16)
	30–39	10 (52)
	40–49	3 (16)
	50–59	3 (16)
Profession	Physiotherapist	6 (31)
	Occupational therapist	4 (21)
	Speech and language therapist	2 (11)
	Nurse	2 (11)
	Psychologist	1 (5)
	Assistant/trainee roles	4 (21)
Years of clinical experience	<1 years	1 (5)
	1–9 years	10 (53)
	10+ years	8 (42)
Years of experience in stroke	<1 year	2 (11)
	1–9 years	13 (68)
	10+ years	4 (21)
Job type^a^	Rotational post	3 (16)
	Static post	16 (84)

^a^Rotational staff spend between 6 and 9 months in one clinical area before rotating to a different clinical area. Static staff remain in one clinical area, in this case community stroke/neuro rehabilitation.

## Theme 1: the team can’t come forever

Therapeutic alliances in these community settings are deliberately built on the premise that *‘…we’re not going to be here forever…’ – P011.* By setting a precedent with stroke survivors that the team can’t come forever clinicians sought to reinforce self-management from the start.

### Sub-theme 1: buy-in

Trust was viewed as important in creating a bond between clinician and stroke survivor, which was considered an important factor to all parties ‘buying-in’ to the relationship, rehabilitation and self-management. This buy-in helped to develop strong alliances with patients and their support networks.

‘*Erm, you need the trust to, sort of, get them on board erm and allow them to see that this is beneficial and in their best interest, ‘cause I know for a lot of patients that can be difficult.’ – P004*

Creating trust was bi-directional. Participants felt it was important to show parts of their personality, as well as demonstrating their professional knowledge, skills and expertise. Informal conversation found common ground, providing incentive for sharing relatable, personal details within professional boundaries. Seeing people in their own environment gave insight into a person's life choices and behaviours, consequently informing how successful rehabilitation and self-management may be. Although this helped with buy-in, it potentially created alliances with unbalanced foundations, assuming a level of dependence and overlooking a person's unique knowledge, skills and values.

### Sub-theme 2: defining expectations

Defining expectations was an important part of developing, maintaining and repairing alliances. Alliances were developed based on clearly communicating expectations of the patient, the professional, the team, potential for recovery and the time-limited nature of rehabilitation. Communication was an important part of defining expectations, particularly finding the balance between being realistic and maintaining hope. Challenging relationships were when expectations were mismatched or unmet. Examples included difficulty adjusting, having high expectations of recovery, or mismatched expectations about self-management. Poorly managed expectations led to alliance fractures which markedly impacted healthcare professionals, reflected in emotionally driven responses grounded in frustration, guilt or a sense of failure. Indicators of alliance fracture were disengagement and confrontation. Strategies to repair alliances included pragmatic and task-based methods, focused on re-negotiating expectations using rehabilitation contracts and outcome measures. These strategies were considered sufficient to maintain an adequate working relationship. Alliances were rarely so fractured that a change of therapist was required.

‘*It's so important that from the start we start laying that foundation of what's realistic and what's expectations… at some point I'm gonna have to walk away and I don't want you to say, you told me that it was gonna be fine and it's not fine.’ – P010.*

### Sub-theme 3: owning roles

To have therapeutic benefits, participants argued that everyone must understand and own their role within therapy. A patient's role is to be an active participant in their recovery and self-management, demonstrated by being engaged and motivated to participate. Barriers to fulfilling this role included low mood, cognitive impairments and communication impairments. Sharing responsibilities with family was considered important in these cases, particularly for self-management; however, this relied on family having capacity to step into the role.

‘*…but that's where you see patients really grow and what I would say then rehab works really well. Erm, is when you've got this dual relationship and clear understanding of responsibilities. And they're not just having rehab done to them.’ – P002.*

The role of the therapist was vast and included being able to conduct their professional responsibilities, working with the wider MDT, managing referrals to other services and third-sector organisations, organising equipment, having difficult conversations, and ending the relationship. Community professionals described many barriers to fulfilling their role, especially dealing with complex social situations. Examples included entering high-risk environments, safeguarding issues and supporting people during mental health crises. When professionals felt unable to fulfil their role, the alliance was considered more challenging.

## Theme 2: therapeutic alliances help and hinder

Therapeutic alliance was viewed as the foundation for rehabilitation and self-management. The ‘rehab relationship’ forms part of the magic of rehabilitation success and in some ways self-management success. However, aspects of the alliance that contribute to successful rehabilitation outcomes may also inhibit self-management.

### Sub-theme 1: power dynamics

Power dynamics were evident throughout the data and this sub-theme has strong relationships with several other sub-themes, as demonstrated in Figure 1. Healthcare professionals described a different power dynamic when working in the community compared to a hospital setting and referenced being ‘*a guest in that person's house’ – P009.* Although participants were aware that the power dynamic shifts when working in the community, the language used to describe working together, e.g. referencing parent–child or teacher–student relationships, *fixing* people, and *giving* skills or empowerment, suggests a complicated power dynamic tipping more towards the therapist. Participants were mindful of creating dependency, yet felt taking control contributed towards the success of rehabilitation. A strong desire to help and a sense of responsibility, rooted in empathy, risked preventing professionals being able to relinquish control in the relationship. Participants felt responsible for providing a positive experience of rehabilitation and recovery in the hope that this would encourage self-management, which then made it difficult to also respect someone's choice to not participate or want to improve.

‘*…I still have this huge feeling of responsibility towards him [laughs]. Erm, so from my end, you know, I've, I've completely bought in and wanna try and work it out. I don't know if it's me doing things to him, you know, he's quite passive in it.’ – P010.*

### Sub-theme 2: goal setting

Goal setting was a key component of the alliance that was important for buy-in and sustained motivation beyond rehabilitation. Experiencing success in the form of a goal increased trust in the professional and their skills, strengthened the alliance and created a sense of safety. Professionals felt this facilitated generating meaningful goals and increased motivation to continue independently after discharge, therefore supporting self-management.

‘*So, starting small and identifying some priorities and some things that are really achievable because then the patient has that experience of success, it's more likely to motivate them to continue.’ – P007.*

Although participants described setting goals in partnership with patients, there were also times when professionals took the lead. Goal setting was an example where the power dynamic complicated the process, particularly where professionals had set self-management goals based on their clinical agenda. As these were not set in partnership they often resulted in non-adherence, with patients re-accessing services and professionals expressing frustration that the ‘self-management plan’ had not been followed.

### Sub-theme 3: supported self-management approaches

Strong alliances were seen as beneficial to influence behaviour during rehabilitation. Goal setting, education and ‘being motivating’ constituted supported self-management approaches. Education was the most common strategy adopted by clinicians and was used interchangeably to facilitate both rehabilitation and self-management. Strong alliances helped the delivery of education and advice, particularly if topics were sensitive or difficult, or when recommending tasks that differ from normal routines. No formal interventions or programmes to support self-management were described by any participant in the study. Only one participant felt education alone was insufficient to influence a person's behaviour.

‘*I mean, you have to provide, you know, education, and we do a lot of that; but just providing education isn't enough.’ – P018.*

The limited knowledge and understanding of support for self-management was seen as problematic across professions. Training needs included relational and psychologically informed practices such as coaching, communication training, motivational interviewing and cognitive-behavioural techniques. NHS organisations streamlining supported self-management approaches across services were a key message. One example was to create ‘self-management champions’ in community teams to raise awareness and maintain momentum.

‘*…but like everything, you need someone to champion…someone in the team to champion [self-management] and be their thing. You need it to be on someone's agenda.’ – P017.*

### Sub-theme 4: it takes a community

Participants recognised the additional burden that self-management could pose after stroke. Alliances during rehabilitation were broadened to include, and potentially expand, a patient's wider support network to facilitate self-management and alleviate the burden. Wider relationships were important for those with severe impairments, such as complex cognitive, communication, physical or mood difficulties. Building awareness of the long-term management of stroke with family and close others supported self-management.

‘*That family member is there for that other 23-hours in a day 7 days a week so, they’re the ones that are doing the looking after, doing the further rehab and I do think that having a good relationship with family members is really important as well.’ – P003*

When a person's network was sparse, developing links with local charities, third-sector organisations, support groups and even using technology supported self-management. The alliance was considered a tool to encourage patients to develop relationships with wider services, in particular, helping patients to feel confident utilising support and expressing their needs to services in the future, consequently improving self-management.

## Theme 3: confusion about what self-management is

Healthcare professionals had different understandings of the term self-management. Self-management was often framed with a focus on the immediate consequences of stroke, such as physical and cognitive impairments, rather than long-term reintegration into life roles, with only one participant suggesting this approach could be problematic.

‘*…we're more focused on can we…within stroke, can we make you back to baseline as in no impairment or can we get you as close as we could be or, unfortunately, do we acknowledge that this is now your new baseline? And all them are very much impairment led, they're not health prevention long-term management led.’ – P005.*

### Sub-theme 1: self-management or self-directed rehab?

Participants reflected that stroke self-management relates to patients taking responsibility for their own health and maintaining their recovery when rehabilitation ends. The tasks of self-management were described as a continuation of the tasks of rehabilitation after discharge from therapy. Participants referenced a process of ‘setting up’ a self-management plan that was in fact ongoing self-directed rehabilitation. As the focus remains on rehabilitation and impairments, an unbalanced alliance is created as healthcare professionals adopt a leading role in determining the tasks that they believe to be important for self-management. This approach minimises a patient's ability to recognise their own skills, resources and values, and plan for their future beyond rehabilitation.

‘*Obviously, I know you’re planning for them in a way but they’re following through what you’ve been doing as a team and then going solo so, yeh…’ - P001*

### Sub-theme 2: self-management is a journey

Time was viewed as an important factor for adjustment after stroke, which for some patients could take years. Participants related this to a grieving process and felt grief could impact rehabilitation at any stage. The need for empathy and flexibility within alliances with patients and families was key to supporting adjustment. Other strategies included rehabilitation breaks/pauses with routes to re-engaging with the team at a later time, and referrals to more appropriate services. Although time was viewed as important in the context of rehabilitation, the importance of timing for self-management was mixed. Some participants felt conversations about self-management should be started early. Whereas others felt self-management was a journey and patients had to accept where they were in order to engage in the process.

‘*…no one's moving on from that impairment level because people aren’t ready to kind of do that because they’re still in this almost like this, this state of grief in a way, um where they can’t progress past that point of, you know, that they think things will return to the way they were…’ – P013*

### Sub-theme 3: self-management is more than tasks and skills

The therapeutic alliance was viewed as a foundation, or building block, for developing and maintaining self-management skills. Participants felt developing self-awareness was key for self-management and perceived their role as supporting patients to understand their stroke impairments and how to manage them long term. Impairment-based approaches gave rise to unbalanced alliances as participants referenced ‘giving skills’ or ‘giving empowerment’, placing healthcare professionals in positions of power. The emotional consequences of stroke for patients and their families were well recognised; however, participants reflected a lack of confidence managing these issues: ‘*dealing with people's emotions is the difficult one.’ – P015.* Having access to neuropsychology was considered most valuable, providing patients with tools to manage complex emotional difficulties, and supporting the wider team with education and advice.

## Discussion

This research is the first to provide an in-depth examination of how therapeutic alliance/working relationships relate to stroke self-management in community settings. The findings reveal several important points about healthcare professionals’ views on therapeutic alliance, and how self-management is supported in practice. Therapeutic alliance is the foundation for rehabilitation and self-management. Participants described developing trusting relationships with patients and their support networks that facilitate successful treatment outcomes. Complicated power dynamics may hinder self-management success as healthcare professionals adopt a leading role in the alliance, setting goals for patients and determining the tasks of self-management. This may stifle patients from developing important self-management skills. Confusion about what self-management is and how it is distinct from self-directed rehabilitation, limits how clinicians support self-management in practice, resulting in impairment-based approaches that replicate the tasks of rehabilitation. These approaches minimise addressing the broader consequences of stroke, such as emotional management and long-term reintegration into life roles.

The findings from this study challenge existing assumptions about the therapeutic alliance in self-management and raise several implications for clinical practice. Healthcare professionals viewed an effective alliance as being constituted of patients buying-in (*bond*), working towards *goals*, and owning their role (*task*) as an active participant in the process, which support the goal, task and bond constructs put forward in Bordin's^6^ early work on therapeutic alliance and more recent work in stroke.^19^ However, in these community settings, less importance was attributed to the bond component. When alliances fractured, direct approaches to repairing them, such as openly communicating a change in the relationship, were not adopted. Outcomes could still be achieved pragmatically, irrespective of whether the bond remained, by re-negotiating the tasks and goals of rehabilitation using contracts and outcome measures. The impact of this on self-management is unclear but previous research suggests poor relationship experiences can potentially impact engagement in future episodes of care,^12^ which would be noteworthy given the long-term consequences of stroke. However, as participants had difficulty separating perceptions of challenging relationships with experiences of difficult rehabilitation, this finding should be interpreted with caution.

In self-management literature, creating an alliance is presented alongside problem solving, decision making, utilising resources and taking action as equally important skills.^5^ In contrast, the current findings highlight that alliance is considered the foundation from which other self-management skills are developed. The findings emphasise the alliance as a mechanism for understanding a person and their needs, establishing meaningful goals, and providing direction for rehabilitation and self-management. The emphasis on person-centredness throughout participants’ narratives is consistent with the literature that argues self-management requires a relationship-based approach.^29^ However, complicated power dynamics may limit patients’ participation in shared goal setting and hinder their opportunities to develop key skills, particularly problem solving and decision making. Focusing on relational practices to support self-management, such as those used in Bridges,^30^ appears to be warranted. As a central component of the alliance and self-management, goal setting has potential to encourage development of important self-management skills. Combining evidence-based interventions, such as ‘Take Charge,’^31^ with enhanced relational practices, may empower more patient control in goal setting and balance the power dynamic. Issues around power and control are well recognised. Mudge at al^32^ describe a complicated balance of clinician's expectations: exercising control whilst also expecting patients to have control over their self-management. The current study's findings demonstrate variations in how self-management is understood based on individual views, aligning with other literature exploring supported self-management in stroke.^3,29,33^ Our findings echo wider literature reinforcing that emotional management is not adequately addressed,^34^ and that professional's descriptions of self-management reflect self-directed rehabilitation and adherence to secondary prevention strategies.^35^

Despite self-management being on clinical and research agendas for over a decade,^34^ there remains a lack of investment in education and training to those delivering services. The findings of this study suggest that moving forward requires three strategies: training and education for clinicians, healthcare professional behaviour change, and organisational support (e.g. self-management champion roles). Despite eight participants in the current study reporting having completed self-management training, clinicians did not use formal interventions or programmes, as recommended in the current stroke guidelines, in clinical practice.^1^ Duncan-Millar et al.'s^29^ work to ascertain what is important in supporting self-management in the community recommends context specific training and guidance based on available resources. Focusing on available resources may be important given the formation of integrated teams and the known disparity of service provision across the United Kingdom. The role of self-management champions also supports work by Duncan-Millar et al.^29^ For self-management support to be implemented effectively, a key focus should be on healthcare professionals’ behaviour. Clinicians must change their behaviours and practices to consider self-management beyond tasks and impairments, and recognise the impact of power.^13^

A strength of this research is the use of qualitative methods to identify the above strategies to improve self-management support in clinical practice. Limitations to our research include healthcare professionals having difficulty describing challenging relationships, which may have been influenced by the interviewer being an ‘insider’.^25^ Power may also have influenced the interviewer–interviewee relationship due to the interviewer's knowledge of self-management and the language used to describe it to participants. The study of alliance should include more than one perspective and by focusing on healthcare professionals, we have thus far only investigated one half of the alliance. An accompanying study exploring stroke survivors views has also been completed.

To conclude, the study findings support the notion that healthcare professionals consider the therapeutic alliance to be a key aspect of rehabilitation and self-management in community stroke services. However, self-management is poorly understood by professionals which limits effective support for stroke survivors. Collaboration between researchers, policy makers, commissioners, healthcare professionals and stroke survivors to conceptualise self-management, may be the first step towards streamlining support strategies in clinical services. Relationship-based training, such as Bridges,^30^ has potential to enhance self-management support in clinical practice. Adequate training combined with champion roles may maintain momentum towards embedding self-management support in routine community stroke care. Future work should also explore how healthcare professionals can change their behaviours and practices to develop alliances where power is shared, despite the challenges and demands of the healthcare setting.

**Clinical messages**

- Clinicians should distinguish between self-directed rehabilitation and self-management.
- The working relationship is an important part of both rehabilitation and the self-management process.
- Support for self-management could be improved through champion roles and staff training e.g. relationship-based self-management approaches.
